# Initiation of eplerenone vs. spironolactone and all-cause mortality in HFrEF: linked database study

**DOI:** 10.1093/ehjcvp/pvag030

**Published:** 2026-04-28

**Authors:** Henrik Svanström, Anders Hviid, Björn Pasternak

**Affiliations:** Department of Data Science and AI in Health, Statens Serum Institut, Artillerivej 5, Copenhagen S 2300, Denmark; Centre for Pharmacoepidemiology, Division of Clinical Epidemiology, Karolinska Institutet, Maria Aspmans gata 30A, 171 64 Solna, Sweden; Department of Data Science and AI in Health, Statens Serum Institut, Artillerivej 5, Copenhagen S 2300, Denmark; Pharmacovigilance Research Center, Department of Drug Design and Pharmacology, University of Copenhagen, Copenhagen, Denmark; Department of Data Science and AI in Health, Statens Serum Institut, Artillerivej 5, Copenhagen S 2300, Denmark; Centre for Pharmacoepidemiology, Division of Clinical Epidemiology, Karolinska Institutet, Maria Aspmans gata 30A, 171 64 Solna, Sweden

**Keywords:** Heart failure, Mineralocorticoid receptor antagonists, Eplerenone, Spironolactone, Comparative effectiveness

## Abstract

**Aims:**

Mineralocorticoid receptor antagonists are recommended as part of guideline-directed medical therapy for heart failure with reduced ejection fraction (HFrEF). The comparative effectiveness of spironolactone and eplerenone, however, remains uncertain.

**Methods and results:**

This was a non-interventional database study using nationwide Danish health registries, 1 January 2020 to 30 June 2025. Patients aged ≥45 years with HFrEF (ejection fraction ≤40%) initiating eplerenone or spironolactone were included. An active-comparator, new-user design and inverse-probability-of-treatment weighting based on propensity scores was used. The primary outcome was all-cause mortality. Secondary outcomes included cardiovascular mortality, heart failure hospitalization, and their composite. Weighted hazard ratios (wHRs) were estimated using Cox proportional hazards models. A total of 4550 patients initiating eplerenone and 6651 initiating spironolactone were included. Median follow-up was 2.3 years. There were 571 deaths in the eplerenone group (5.0 per 100 person-years) and 1168 in the spironolactone group (7.0 per 100 person-years). A lower risk of death was observed with eplerenone [wHR, 0.83; 95% confidence interval (CI), 0.75–0.93], corresponding to an absolute rate difference of −1.10 per 100 person-years (95% CI, −0.50 to −1.70). For secondary outcomes, wHRs were 0.89 (95% CI, 0.81–0.98) for cardiovascular death or heart failure hospitalization, 0.79 (95% CI, 0.67–0.92) for cardiovascular death, and 0.97 (95% CI, 0.87–1.09) for heart failure hospitalization. Effect estimates were attenuated in per-protocol analyses.

**Conclusion:**

In this large nationwide study, initiation of eplerenone was associated with lower risks of all-cause and cardiovascular mortality compared with spironolactone, with findings suggesting a contribution of better treatment persistence.

## Graphical Abstract

**Graphical Abstract:**
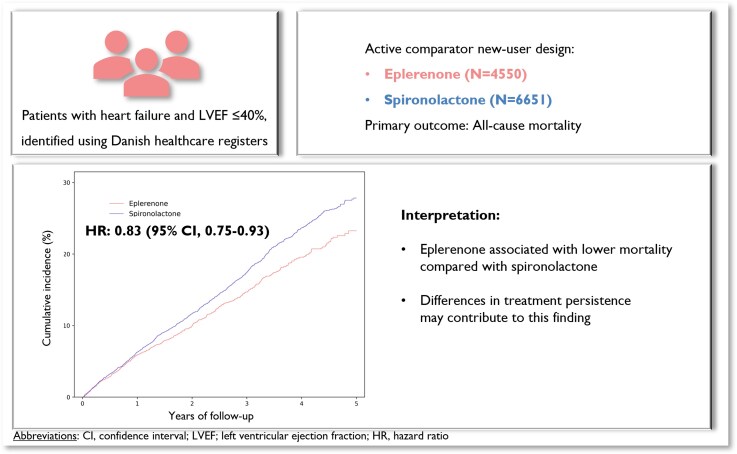

Mineralocorticoid receptor antagonists (MRAs) are recommended for all patients with heart failure with reduced ejection fraction (HFrEF), alongside renin–angiotensin system inhibitors, beta-blockers, and sodium-glucose cotransporter 2 (SGLT2) inhibitors.^1,2^

In the seminal RALES and EMPHASIS-HF trials among patients with HFrEF, spironolactone and eplerenone—the two MRAs currently recommended in clinical heart failure guidelines—demonstrated similar relative risk reductions in all-cause mortality, compared with placebo.^3,4^ While both of these agents act by antagonizing the mineralocorticoid receptor, they also differ in important pharmacologic properties.^5^ Compared with spironolactone, eplerenone is more selective, which may reduce the risk of hormonal side effects such as gynaecomastia. Both agents require regular monitoring of renal function and serum potassium, particularly when used in combination with renin–angiotensin system inhibitors.

To date, however, no large-scale head-to-head trials have evaluated the comparative effectiveness of spironolactone vs. eplerenone in patients with HFrEF, and their relative clinical benefits in routine care remain uncertain. The seminal trials were conducted more than a decade apart and enrolled distinct patient populations receiving different background therapies, limiting the interpretability of indirect comparisons.^3,4^ While small randomized trials and observational studies have compared spironolactone and eplerenone in HFrEF and suggest lower risks of mortality, gynaecomastia, and treatment withdrawal in favour of eplerenone, the available data remain limited, conflicting, and methodologically heterogenous.^6–9^

Using a nationwide clinical database in Denmark,^10^ this study compared the effectiveness of eplerenone to spironolactone in patients with HFrEF.

## Methods

This was a non-interventional database study conducted in Denmark using data from the Danish Heart Failure Registry (DHR), linked with national health and administrative registers, covering the period from 1 January 2020, to 30 June 2025. The start of the study period was chosen to align with evolving guideline-directed medical therapy for HFrEF, coinciding with the broader integration of four core medication classes —including SGLT2 inhibitors and the recommendation of MRA therapy for all patients with HFrEF— as well as the wider adoption of eplerenone in Denmark.

The study population comprised patients aged 45 years or older with a diagnosis of HFrEF, defined as a left ventricular ejection fraction (LVEF) ≤ 40%, recorded up to 30 June 2024. For inclusion, patients were required to have initiated spironolactone or eplerenone from 1 January 2020, to 31 December 2024. Relevant Anatomical Therapeutic Chemical (ATC) classification codes are listed in Supplementary material online, *Table S1*.

Patients with HFrEF were identified using the DHR, which captures all first-time, specialist-confirmed cases of heart failure in Denmark. The registry employs International Statistical Classification of Diseases, 10th Revision (ICD-10) codes, including I11.0, I13.0, I13.2, I42.0, I42.6, I42.7, I42.9, I50.0, I50.1, and I50.9. Reporting to the DHR is mandatory for all hospital departments treating in- or outpatients with incident heart failure.

An active comparator new-user design was applied to emulate a comparative trial. The index date was defined as the date of first prescription for either agent following diagnosis of heart failure. Patients were excluded if they had received any MRA during the 2 years prior to the index date. Additional exclusion criteria included prior heart transplantation, end-stage illness, substance misuse, severe renal dysfunction (end-stage kidney disease or renal transplantation), eGFR <30 mL/min/1.73 m^2^, or current nursing home residence. Full definitions for these criteria are provided in Supplementary material online, *Table S2*.

Drug exposure data were obtained from the Danish National Prescription Registry (DNPR),^11^ which captures all pharmacy-dispensed medications nationwide, including ATC codes and dates of dispensation. Vital status and date of death were ascertained from the Civil Registration System (CRS),^12^ a continuously updated registry with practically complete national coverage. Cardiovascular deaths were identified through the Danish Register of Causes of Death (CDR),^13^ while hospitalizations for heart failure were captured via the National Patient Register (NPR).^14^ The NPR contains detailed information on all inpatient and outpatient hospital visits in Denmark, including ICD-10–coded diagnoses, dates, and care settings. The CDR uses ICD-10 codes recorded on official death certificates. The composite outcome was defined as the earliest event of either cardiovascular death or heart failure hospitalization. Full outcome definitions are listed in Supplementary material online, *Table S3*.

To address baseline imbalances between treatment groups, inverse-probability of treatment weighting (IPTW) based on propensity scores was applied, yielding an estimate of the average treatment effect (ATE) for eplerenone vs. spironolactone. Propensity scores were estimated through logistic regression including all variables listed in *Table 1*. Covariates were drawn from several national data sources: heart failure-related clinical data from the DHR (e.g. LVEF, NYHA class);^10^ demographic characteristics from the CRS;^12^ attained education from Statistics Denmark; medical history, hospital procedures, and healthcare utilization from the NPR;^14^ outpatient prescription fills from the DNPR; and biomarkers from the Danish Register of Laboratory Results for Research.^15^  Supplementary material online, *Table S4* provides the full covariate definitions.

**Table 1:** Baseline characteristics after propensity score weighting

Characteristic	Eplerenone	Spironolactone	SMD
*N*	4550	6551	
Male sex	70	70	0.01
Age (years), mean (SD)^a^	70.2 (10.5)	70.2 (10.7)	
Age (years), categorized			
45–49	4	4	<0.01
50–54	6	6	<0.01
55–59	10	10	<0.01
60–64	12	12	<0.01
65–69	14	14	<0.01
70–74	17	17	<0.01
75–79	18	18	<0.01
80–84	13	13	<0.01
≥85	7	7	0.01
Country of birth			
Denmark	93	93	0.01
Rest of Europe	5	5	0.01
Outside Europe	2	3	<0.01
Civil status			
Not married	47	47	0.01
Married	53	53	0.01
Attained education			
Primary school	32	32	<0.01
Secondary school and vocational training	45	45	<0.01
Tertiary education	17	17	<0.01
Education: Missing	6	6	<0.01
Calendar year ^a^			
2020	11	20	
2021	20	20	
2022	26	19	
2023	28	24	
2024	15	17	
Lifestyle health factors			
Alcohol consumption^b^			
Below or at recommendations	86	86	<0.01
Above recommendations	11	11	<0.01
Missing	2	2	<0.01
Smoking			
Never	33	33	<0.01
Current	25	25	<0.01
Past	40	40	<0.01
Missing	2	2	<0.01
HF characteristics			
LVEF (%), mean (SD)^a^	28.7 (8.4)	28.7 (8.4)	
LVEF (%), categorized			
35–40	36	36	<0.01
30–34	21	21	<0.01
25–29	16	15	0.01
<25	28	28	<0.01
NYHA classification			
I	12	12	<0.01
II	71	71	<0.01
III	16	16	<0.01
IV	1	1	<0.01
Missing	0	1	0.02
Hospitalized at the time of first HF diagnosis	50	51	0.01
Days since first HF diagnosis, median (IQR)^a^	50.0 (13.0–147.0)	54.0 (12.0–152.0)	
Days since first HF diagnosis, categorized			
<90	63	63	0.01
90–179	15	15	0.01
180–364	7	6	<0.01
≥365	15	16	0.02
Renal function			
eGFR (mL/min/1.73 m^2^), mean (SD)^a^	72.7 (17.2)	72.7 (17.5)	
eGFR (mL/min/1.73 m^2^), categorized			
≥90	19	19	<0.01
60–89	55	55	<0.01
45–59	20	19	<0.01
30–44	5	5	0.01
Missing	1	1	0.01
Medical history in the previous, 10 years			
Acute coronary syndrome	20	20	<0.01
Ischaemic heart disease	29	29	0.01
Coronary revascularization in the previous year	15	15	0.01
Other cardiac surgery or invasive procedure in the previous years	4	4	<0.01
Cardiomyopathy	12	12	<0.01
Valve disorder	9	9	<0.01
Stroke	8	8	<0.01
Other cerebrovascular disease	6	7	<0.01
Atrial fibrillation	33	33	<0.01
Other arrythmia	18	18	<0.01
Implantable cardioverter defibrillator	2	2	<0.01
CRT with pacemaker	1	1	<0.01
Peripheral arterial disease	9	9	<0.01
Kidney disease diagnosis	7	7	0.01
Diabetes complications	8	8	<0.01
COPD	9	10	0.01
Other lung disease	10	11	<0.01
Venous thromboembolism	5	5	<0.01
Cancer (excl. non-melanoma skin cancer)	14	14	<0.01
Liver disease	2	2	<0.01
Osteoporosis	10	10	0.01
Fracture in the previous year	3	3	0.01
Alcohol-related disorders	3	3	<0.01
Health care utilization			
HF hospitalization in the previous 0–29 days	30	30	<0.01
HF hospitalization in the previous 30–364 days	26	25	0.01
Other CV hospitalization in the previous 0–29 days	12	12	<0.01
Other CV hospitalization in the previous 30–364 days	29	29	0.01
Other hospitalization in the previous 0–29 days	17	17	0.01
Other hospitalization in the previous 30–364 days	43	43	<0.01
HF outpatient hospital contact in the previous 0–29 days	67	66	0.02
HF outpatient hospital contact in the previous 30–364 days	45	44	0.01
Other CV outpatient hospital contact in the previous 0–29 days	12	12	0.01
Other CV outpatient hospital contact in the previous 30–364 days	25	25	<0.01
Other outpatient hospital contact in the previous 0–29 days	38	38	<0.01
Other outpatient hospital contact in the previous 30–364 days	73	73	<0.01
Home nursing care in the previous 365 days	22	23	0.01
Prescription drug use in the previous 365 days			
Loop diuretic	67	67	<0.01
Other diuretic	11	11	<0.01
ACEi	61	61	0.01
ARB	31	31	<0.01
ARNI	5	5	0.01
Beta-blocker	85	85	0.01
SGLT2-inhibitor	42	41	0.02
Calcium-channel blocker	24	24	<0.01
Digoxin	10	10	0.01
Nitrate	12	12	0.01
Antiarrhythmic drugs	7	7	<0.01
Platelet inhibitors	46	46	<0.01
Anticoagulants	41	41	<0.01
Lipid lowering drug	61	61	<0.01
Metformin	16	16	<0.01
DPP4 inhibitor	2	2	<0.01
GLP-1 RA	6	5	<0.01
Insulin	6	6	<0.01
Other antidiabetic	1	1	<0.01
Antidepressant	13	13	0.01
Antipsychotic	3	3	<0.01
Anxiolytic, hypnotic, or sedative	12	13	0.01
Beta-2 agonist inhalant	18	18	<0.01
Anticholinergic inhalant	4	4	0.01
Glucocorticoid inhalant	11	11	<0.01
Oral glucocorticoid	10	11	<0.01
Opioid	16	16	0.01
Number of different drugs used in the previous 365 days			
<10	34	34	0.01
10–14	39	39	<0.01
15–19	19	19	0.01
≥20	8	8	<0.01

Values are percentages unless otherwise stated.

COPD, chronic obstructive pulmonary disease; CRT, cardiac resynchronization therapy; CV, cardiovascular; DPP4, dipeptidyl peptidase 4; eGFR, estimated glomerular filtration rate; GLP-1 RA, glucagon-like peptide-1 receptor agonist; LVEF, left ventricular ejection fraction; MRA, mineralocorticoid receptor antagonist; NYHA, New York Heart Association; SGLT2, sodium-glucose co-transporter 2

^a^Not included in propensity score model.

^b^Recommended limit was defined as ≤21 units per week among men and ≤14 units per week among women

For the variables with missing data—specifically attained education, NYHA class, eGFR, alcohol use, and smoking (≤6% missing for all covariates)—missingness was handled by incorporating separate missing categories in the propensity score model.^16^ Patients with propensity scores falling outside the region of common support for the treatment groups were excluded. The IPTW weights were truncated at the 1st and 99th percentiles to reduce the impact from extreme values.^17^ Covariate balance following weighting was evaluated using standardized mean differences (SMDs), with <0.1 indicating acceptable balance.

Treatment status was defined using the observational analogue of an intention-to-treat approach: patients remained in their initially assigned treatment group for the full duration of follow-up, regardless of prescription refills, with censoring only at crossover to the alternative agent.

In this framework, treatment discontinuation was considered a post-initiation event reflecting tolerability and persistence of the initially selected therapy. Follow-up was therefore retained after discontinuation to capture these effects. In contrast, crossover to the alternative MRA represents receipt of the competing intervention and was censored to avoid mixing exposure between treatment strategies and to preserve a clear analytic contrast. This approach allowed estimation of the effect of initiating alternative MRA treatment strategies in routine care.

To evaluate how the results varied under alternative exposure definitions, two complementary analyses of the primary outcome were conducted. First, a per-protocol analysis was performed: each dispensed tablet was assumed to represent one day of supply, and a 30-day grace period was added after the expected duration of each prescription to account for refill irregularities. As with all prescription-based studies, dispensed medications were used as a proxy for actual drug use, and adherence beyond prescription fills could not be directly assessed. Follow-up was censored at the end of this grace period if treatment was discontinued. Inverse probability of censoring weights were applied to adjust for potential selection bias. Second, an intention-to-treat analysis was conducted in which patients remained in their original treatment group throughout follow-up, without censoring in the case of crossover. Together, these complementary analyses allowed the assessment of how treatment discontinuation and persistence influenced the estimated treatment effects, by comparing estimates under strategies that continue vs. censor follow-up after treatment discontinuation

In the main analysis, follow-up began on the index date and continued until the occurrence of an outcome event or censoring due to emigration or end of the study period (30 June 2025). For the composite outcomes, the event date was defined as the date of the first occurrence of any component within the composite.

Hazard ratios (HRs) for the primary and secondary outcomes were estimated using proportional hazards regression with days since the index date as the time scale. To account for non-independence arising from weighting, robust sandwich variance estimators were applied in all analyses. Absolute rate differences were estimated using weighted Poisson regression, with variances adjusted using generalized estimating equations. For descriptive purposes, treatment discontinuation was defined as a gap of more than 30 days without a dispensed tablet, assuming one tablet per day; this definition was consistent with that used in the complementary per-protocol analysis.

Subgroup analyses were conducted by calculating propensity scores and applying weighting separately within each subgroup. The specific definitions used to define each subgroup are listed in Supplementary material online, *Table S5*. Effect modification was assessed using Wald tests for interaction, with statistical significance defined as a *P*-value <0.05.

All statistical analyses were conducted using SAS software version 9.4.

The study was conducted in accordance with national regulations governing the use of health data for research. In Denmark, ethics committee approval is not required for register-based studies.

## Results

**Figure 1:**
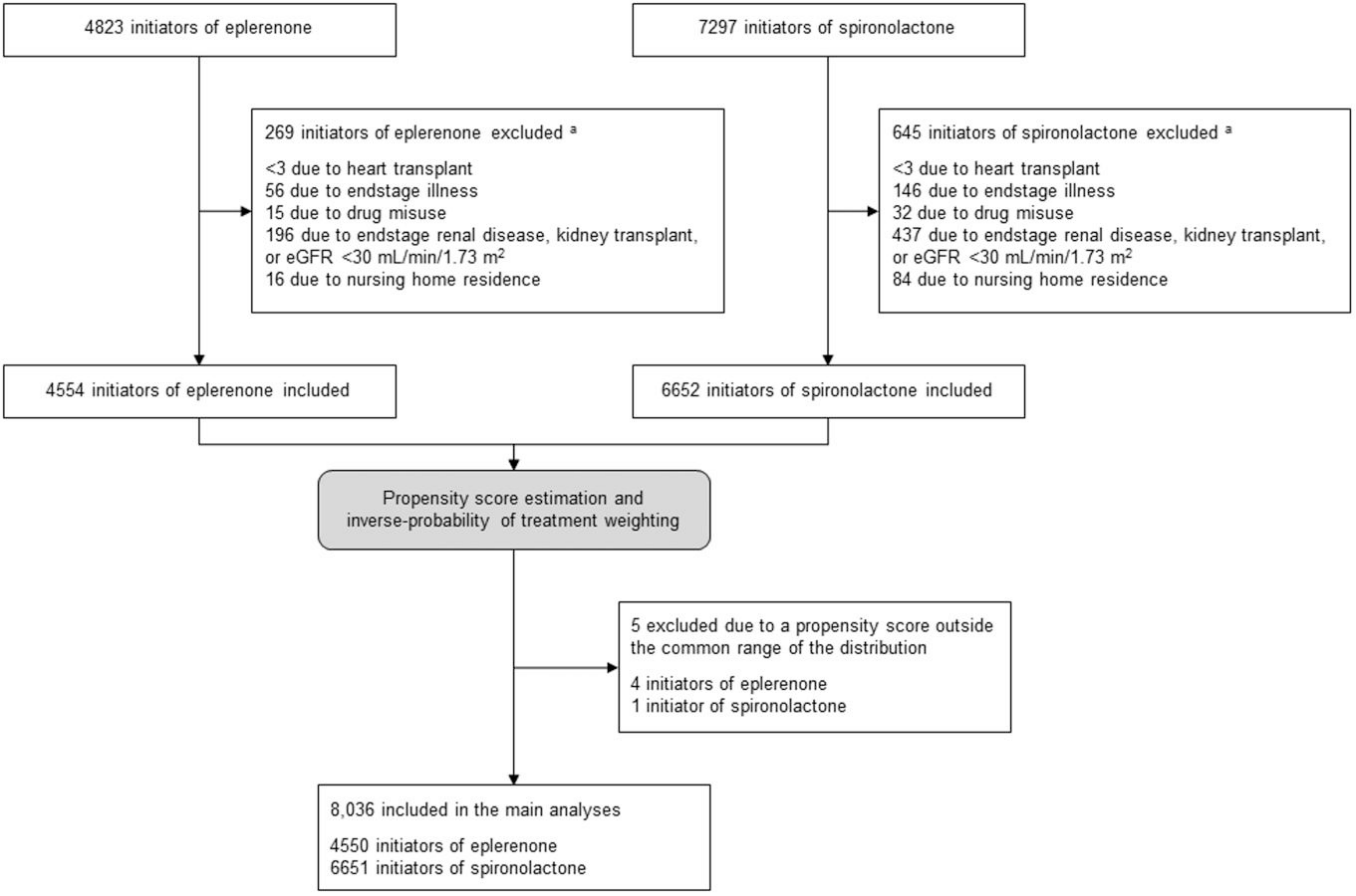
Inclusion of initiators of eplerenone and spironolactone. ^a^Patients could be excluded for multiple reasons.

After applying exclusion criteria and removing patients with non-overlapping propensity scores, 4550 patients initiating eplerenone and 6651 initiating spironolactone were included in the analysis (*Figure 1*).

Baseline characteristics before and after weighting are presented in Supplementary material online, *Table S6* and *Table 1*, respectively. Odds ratios from the propensity score model are shown in Supplementary material online, *Table S7*. After weighting and truncation, the mean stabilized weight was 1.00 and the maximum was 1.91. Covariates were well balanced between groups, with all SMDs below 0.1 (*Table 1*).

In both groups, 70% of patients were male, and the mean age at baseline was 70.2 years. The median time from heart failure diagnosis to MRA initiation was 50 days (IQR, 13–147) in the eplerenone group and 54 days (IQR, 12–152) in the spironolactone group.

In the primary outcome analysis, median follow-up was 2.3 years (IQR, 1.5–3.4) in the eplerenone group and 2.3 years (IQR, 1.3–3.7) in the spironolactone group. The rate of treatment discontinuation was 64.5 per 100 person-years among eplerenone users and 79.1 per 100 person-years among spironolactone users (incidence curve shown in Supplementary material online, *Figure S1*). To further contextualize treatment modification patterns, a *post hoc* descriptive analysis of laboratory abnormalities among patients who discontinued treatment was conducted. Specifically, among patients who discontinued spironolactone, 5.6% had at least one plasma potassium measurement ≥5.5 mmol/L during follow-up prior to treatment discontinuation, compared with 3.1% among those who discontinued eplerenone. The corresponding proportions with eGFR <30 mL/min/1.73 m^2^ were 12.5% and 8.4%, respectively. Treatment crossover occurred in 4% of eplerenone users and 10% of spironolactone users.

**Figure 2:**
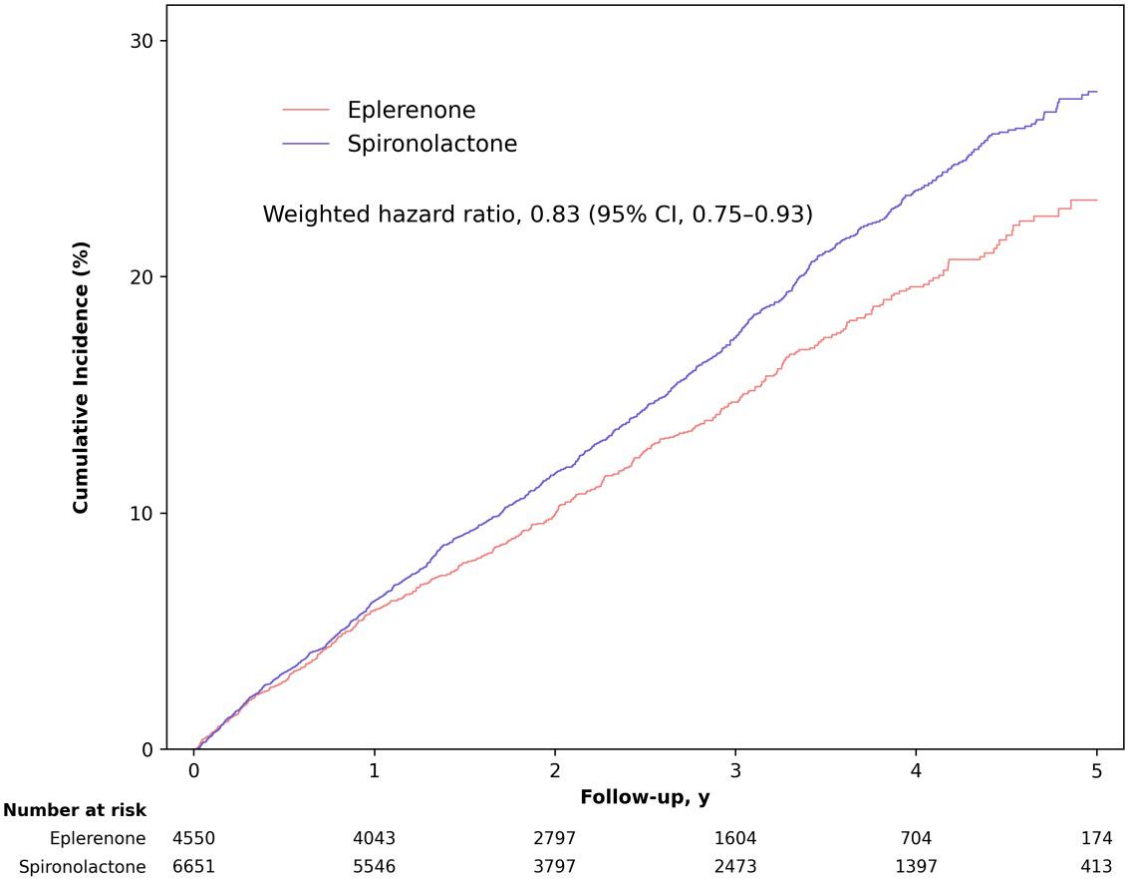
Weighted ^a^cumulative incidence curve for the primary outcome of all-cause mortality. The cumulative incidence curve was truncated at 5 years due to decreasing numbers of patients at risk and outcome events. The maximal follow-up in the study was 5.5 years. ^a^Inverse probability of treatment weighting according to propensity score.

The primary outcome results are shown in *Figure 2*. There were 571 deaths in the eplerenone group (crude rate 5.0 per 100 person-years) and 1168 in the spironolactone group (7.0 per 100 person-years). The propensity-score weighted cumulative incidence of all-cause mortality at 5 years was 23.3% for eplerenone and 27.8% for spironolactone. Eplerenone was associated with a weighted HR (wHR) of 0.83 (95% CI, 0.75–0.93) and weighted absolute rate difference of −1.10 deaths per 100 person-years (95% CI, −0.50 to −1.70), compared with spironolactone.

The results for the secondary outcomes are shown in *Table 2*, with cumulative incidence curves presented in Supplementary material online, *Figures S2*–*S4*. The wHR associated with initiating eplerenone, compared with spironolactone, was 0.89 (95% CI, 0.81–0.98) for the composite of cardiovascular death or heart failure hospitalization, 0.79 (95% CI, 0.67–0.92) for cardiovascular death, and 0.97 (95% CI, 0.87–1.09) for heart failure hospitalization.

**Table 2:** Results for the secondary outcomes

Outcome	Eplerenone (*n* = 3195)		Spironolactone (*n* = 4841)		Hazard ratio (95% CI)
	Events	Rate per 100 p-y	Events	Rate per 100 p-y	
CV death or HF hospitalization	672	6.3	1228	8.0	0.89 (0.81–0.98)
CV death	247	2.2	534	3.2	0.79 (0.67–0.92)
HF hospitalization	518	4.8	862	5.6	0.97 (0.87–1.09)

CI, confidence interval; CV, cardiovascular; p-y, person-years; HF, heart failure.

**Figure 3:**
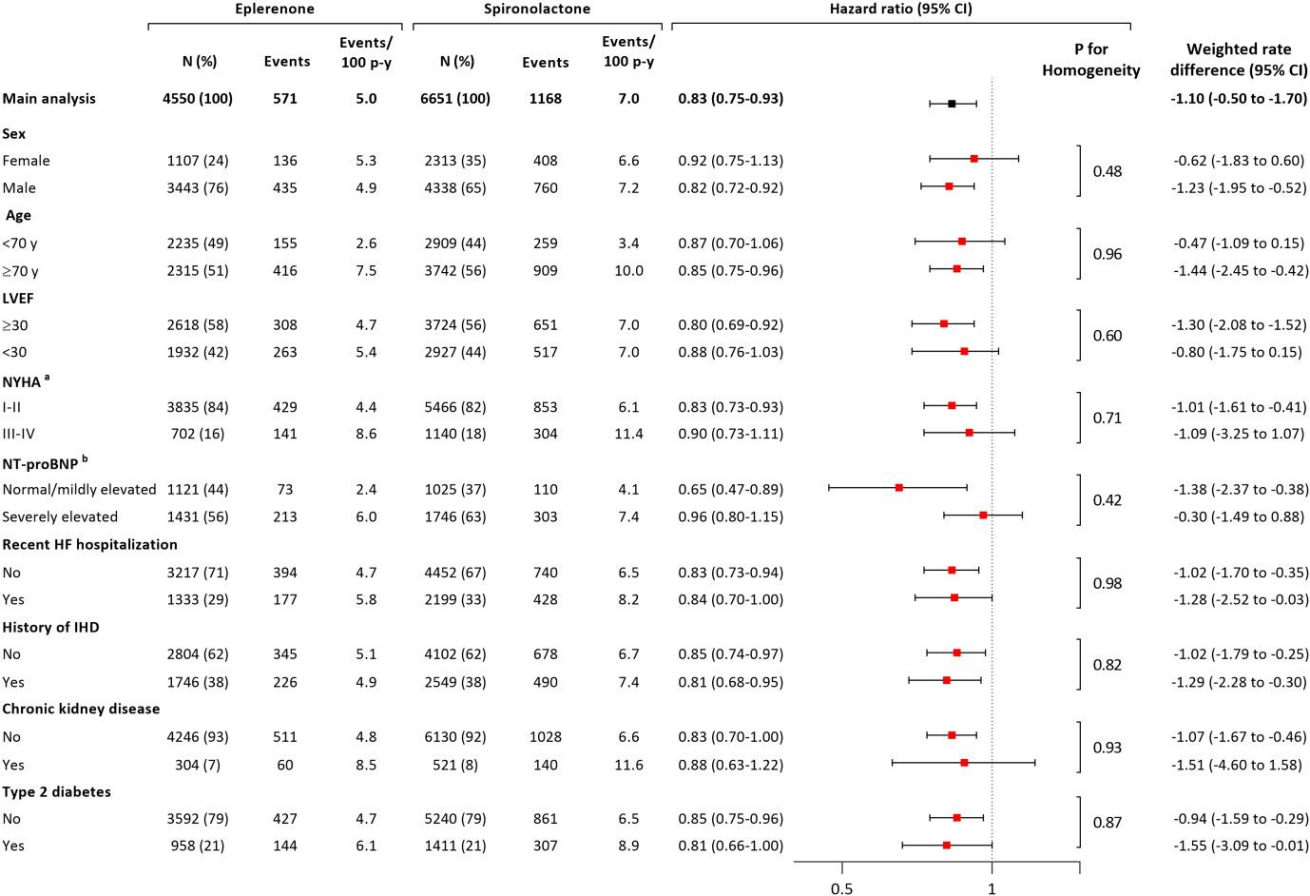
Results of subgroup analyses of the primary outcome. Abbreviations: CI, confidence interval; CKD, chronic kidney disease; HF, heart failure; IHD, ischaemic heart disease; LVEF, left ventricular ejection fraction; NT-proBNP, N-terminal prohormone of brain natriuretic peptide; NYHA, New York Heart Association; p-y, person-years. ^a^Inverse probability of treatment weighting according to propensity score; ^b^Patients with missing information on NYHA-category were not included in this subgroup analysis; ^c^Patients with missing information on NT-proBNP were not included in this subgroup analysis

The results for the subgroup analyses of the primary outcome are shown in *Figure 3*. While the absolute rates varied substantially across several subgroup strata, no statistically significant treatment effect heterogeneity was observed, including by chronic kidney disease status, ejection fraction level, or NYHA class.

The results of complementary analyses of the primary outcome are shown in Supplementary material online, *Table S7*. When using a per-protocol approach, which accounted for treatment discontinuation, the wHR was 0.96 (95% CI, 0.81–1.13) for eplerenone (4.1 deaths per 100 person-years), compared with spironolactone (5.4 deaths per 100 person-years) (incidence curves in Supplementary material online, *Figures S5*). Using an ITT approach without censoring at crossover resulted in a wHR of 0.91 (95% CI, 0.83–1.01) for eplerenone (5.2 deaths per 100 person-years), compared with spironolactone (6.6 deaths per 100 person-years).

## Discussion

In this real-world study of patients with HFrEF, treatment with eplerenone was associated with a statistically significant 17% lower risk of all-cause mortality compared with spironolactone, corresponding to 1.1 fewer deaths (95% CI, 0.5–1.7) per 100 person-years. This supports the relative effectiveness of eplerenone over spironolactone. Notably, treatment discontinuation was frequent in both groups and occurred less often among patients initiating eplerenone. This pattern is consistent with the more favourable tolerability profile of eplerenone and suggests that differences in treatment persistence may, at least in part, explain the observed differences in clinical outcomes. Results were consistent across all investigated key subgroups. For secondary outcomes, eplerenone was associated with a lower risk of cardiovascular death or heart failure hospitalization and cardiovascular death, but not for heart failure hospitalization alone.

The seminal RALES trial demonstrated that spironolactone reduced all-cause mortality by 30% (HR 0.70; 95% CI, 0.60–0.82) among patients with LVEF ≤35%, NYHA class III–IV symptoms, and background treatment with an angiotensin converting enzyme inhibitor and loop diuretic.^3^ Similarly, the EMPHASIS-HF trial,^4^ which enrolled patients with LVEF ≤35% and NYHA class II symptoms receiving a renin–angiotensin system inhibitor and beta-blocker, reported a 24% reduction in mortality with eplerenone (HR 0.76; 95% CI, 0.62–0.93), compared with placebo. These trials were conducted in different eras, enrolled distinct populations with varying background therapies, and should therefore not be compared directly.

There is a paucity of high-quality large-scale analyses directly comparing MRAs in HFrEF. A small, single-centre randomized trial (*n* = 142), albeit with limited methodological detail, reported that eplerenone was associated with lower all-cause mortality (HR 0.64; 95% CI, 0.44–0.93) and cardiovascular mortality (HR 0.53; 95% CI, 0.34–0.82), but not with a reduction in the composite of cardiovascular death or heart failure hospitalization (HR 0.95; 95% CI, 0.73–1.27), compared with spironolactone.^8^ In a small (*n* = 586), single-centre observational study from Spain,^6^ eplerenone was associated with lower risks of all-cause mortality (HR 0.67; 95% CI, 0.47–0.95) and cardiovascular mortality compared with spironolactone but not with heart failure hospitalization. Key limitations, however, included the lack of a clearly defined treatment initiation date relative to follow-up start, raising concerns about selection and potential immortal time bias. In addition, the use of a propensity score–matched (average treatment effect on the treated) design excluded nearly 20% of eplerenone users due to lack of suitable matches—limiting confidence in the representativeness and interpretability of the findings. A second observational study in Denmark found no significant association between eplerenone use and mortality (HR 0.93; 95% CI, 0.67–1.30) although it relied solely on diagnostic codes and lacked clinical information, limiting the ability to adjust for disease severity or treatment context.^7^ Furthermore, the study period (2016–2020) preceded the updated clinical guidelines recommending MRAs for all patients with HFrEF and the broader adoption of eplerenone in Danish heart failure care; during that time, eplerenone was typically reserved for patients who had experienced adverse effects from spironolactone, as reflected in the markedly skewed sex distribution among eplerenone users (91% male). A head-to-head meta-analysis combining the observational studies with the small randomized trial reported a lower risk of all-cause mortality (HR 0.74, 95% CI, 0.59–0.94) and cardiovascular mortality (HR 0.54, 95% CI, 0.39–0.74) for eplerenone compared with spironolactone.^9^

While collectively suggestive of a benefit with eplerenone, the previously available evidence suffers from important limitations in design, generalizability, or analytical clarity. The present analysis substantially expands the evidence base by evaluating a large, contemporary, nationwide population of patients with HFrEF initiating MRA therapy under current guideline-directed management. Leveraging granular clinical and administrative data and applying robust methods to account for confounding, this study provides a real-world estimate of comparative effectiveness in a broad population drawn from routine clinical practice. The findings indicate a clinically meaningful difference in favour of eplerenone.

The mechanism underlying this difference, if true, remains uncertain. Nevertheless, there are important biological distinctions between the two agents that appear to translate into differences in tolerability and adherence. While both spironolactone and eplerenone act on the renal system and are associated with a risk of hyperkalaemia—particularly when combined with renin–angiotensin system inhibitors—this risk appears broadly similar between the two agents.^1,5,18^ In contrast, eplerenone is more selective for the mineralocorticoid receptor and is associated with a lower incidence of hormonal adverse effects, such as gynaecomastia.^2,5^ Observational data from multiple settings have consistently suggested higher treatment persistence with eplerenone compared with spironolactone.^6,7,19^ In the present study, the rate of treatment discontinuation was 64.5 per 100 person-years among eplerenone users and 79.1 per 100 person-years among spironolactone users (see Supplementary material online, *Figure S1*). Importantly, the mortality benefit observed in the main analysis was almost fully attenuated in the per-protocol analysis, where patients were censored at treatment discontinuation. The delayed separation of incidence curves further supports the interpretation that differences in treatment persistence—and by extension tolerability—may have played a central role in the observed effect. This suggests that among patients who tolerate and persist with therapy, spironolactone and eplerenone may offer similar effectiveness. However, in real-world practice, outside controlled protocols, overall effectiveness will also depend on tolerability and treatment persistence, both of which appear higher with eplerenone.

Newer non-steroidal MRAs, such as finerenone, have been developed to provide more selective mineralocorticoid receptor antagonism with fewer off-target effects and have demonstrated cardiovascular benefit in selected populations. Whether improved tolerability of these agents translates into better treatment persistence and outcomes in routine heart failure care remains to be established.

The study had several strengths. First, it leveraged high-quality, nationwide data sources, including a specialist-confirmed heart failure registry linked to comprehensive prescription and mortality records, enabling robust identification of patients with HFrEF and outcome ascertainment. Second, granular clinical information such as left ventricular ejection fraction, NYHA class, renal function, and biomarker data allowed for detailed adjustment for clinical confounders using propensity score weighting. Third, the study design emulated a real-world treatment decision between eplerenone and spironolactone, using an active comparator new-user design and modified intention-to-treat framework. Fourth, the inclusion of per-protocol analysis and treatment persistence data, enabled exploration of persistence-related mechanisms. Finally, the study was conducted in a contemporary treatment era in which MRAs are recommended for all patients with HFrEF, increasing the relevance of findings to current clinical practice.

Several limitations should also be noted. First, new prescriptions of the study drugs were used as a proxy for the emulated treatment decision, which may not fully capture the actual initiation of therapy. In addition, in the complementary per-protocol analysis, adherence beyond prescription fills could not be directly assessed, and information on achieved dose or dose titration over time was not available. Assuming any misclassification was non-differential, this would likely bias the results towards the null. Second, although the main analysis showed a statistically significant 17% reduction in all-cause mortality, limited precision in some subgroup analyses may have prevented detection of modest effect differences. Third, no data were available on factors such as blood pressure control or non-pharmacologic interventions (e.g. exercise, weight management). Fourth, although the proportion of missing covariate data was low, residual confounding related to patterns of missingness cannot be excluded. Finally, despite multiple efforts to maximize internal validity, as with any non-randomized study, the possibility of unmeasured confounding cannot be excluded. The observed association was moderately robust to unmeasured confounding; the *E*-value for the primary estimate was 1.70 (1.36 for the confidence interval limit). Patients with HFrEF often have complex clinical profiles and care needs, which may influence treatment selection, tolerability, and persistence in routine care. However, the groups were well-balanced on measured baseline characteristics even before propensity score weighting, suggesting near-equipoise in the clinical use of these two agents.

In this large database study emulating a comparative trial, the use of eplerenone was associated with a significant reduction in mortality compared with spironolactone among patients with HFrEF. This difference appeared to be driven by better treatment persistence with eplerenone, consistent with its more favourable tolerability profile. These findings expand on the evidence supporting the comparative effectiveness of eplerenone over spironolactone.

## Supplementary Material

pvag030_Supplementary_Data

## Data Availability

Data from the Danish nationwide registers may be obtained from a third party and are not publicly available. According to Danish data protection regulations, data cannot be shared publicly.

## Transparency

The lead author (the manuscript’s guarantor) affirms that the manuscript is an honest, accurate, and transparent account of the study being reported; that no important aspects of the study have been omitted; and that any discrepancies from the study as planned (and, if relevant, registered) have been explained.
