# Supplementary material for: Initiation of eplerenone vs. spironolactone and all-cause mortality in HFrEF: linked database study
Source: Eur Heart J Cardiovasc Pharmacother. 2026 Apr 28;12(4):282–91. doi: 10.1093/ehjcvp/pvag030 (PMC13367240; doi:10.1093/ehjcvp/pvag030)

**Supplement:

Initiation of Eplerenone vs Spironolactone and All-cause Mortality in HFrEF:**

**Linked Database Study**

**Table of contents**

[Supplementary Table S1. Inclusion and exclusion criteria 1](#_Toc213851745)

[Supplementary Table S2. Definitions of the primary and secondary outcomes 2](#_Toc213851746)

[Supplementary Table S3. Definitions of covariates and other baseline characteristics 3](#_Toc213851747)

[Supplementary Table S4. Subgroup definitions 8](#_Toc213851748)

[Supplementary Table S5. Baseline characteristics before propensity score weighting. 9](#_Toc213851749)

[Supplementary Table S6. Odds ratios from the propensity score model 14](#_Toc213851750)

[Supplementary Figure S1. Weighted incidence curve for treatment discontinuation in the analysis of the primary outcome 18](#_Toc213851751)

[Supplementary Figure S2. Weighted cumulative incidence curve for cardiovascular death or heart failure hospitalization 19](#_Toc213851752)

[Supplementary Figure S3. Weighted cumulative incidence curve for cardiovascular death 20](#_Toc213851753)

[Supplementary Figure S4. Weighted cumulative incidence curve for heart failure hospitalization 21](#_Toc213851754)

[Supplementary Table S7. Sensitivity analyses of the primary outcome 22](#_Toc213851755)

[Supplementary Figure S5. Incidence curve for the primary outcome using per-protocol analysis 23](#_Toc213851756)

# Supplementary Table S1. Inclusion and exclusion criteria

| Criterion | Definition ^a^ | Codes | Source |
| --- | --- | --- | --- |
| Inclusion criteria | | | |
| Treatment with spironolactone or eplerenone | Prescription for spironolactone or eplerenone after first-time specialist care HF diagnosis | Spironolactone: C03DA01 | DNPR |
|  |  | Eplerenone: C03DA04 |  |
| Age 45 y or older | - | N/A | CRS |
| LVEF ≤40% | - | N/A | DHR |
| Exclusion criteria | | | |
| Previous treatment with any MRA | Prescription for any MRA in the previous 730 days before the index date | ATC: C03DA | DNPR |
| Heart transplant | - | NCSP: KFQ | NPR |
| End-stage renal disease, kidney transplant, or eGFR<30 mL/min/1.73m2 | Dialysis (in the previous 365 d), kidney transplant, or eGFR<30 mL/min/1.73m2 (in the previous 180 d) | ICD-10: Z49.x, Z94.0, Z99.2  NCSP: BJFD, BJFZ, KKAS | NPR,  RLRR |
| End-stage illness | Diagnosis or treatment for conditions associated with severe health deterioration and high risk of mortality, including severe malnutrition, dementia, decubitus ulcer, gangrene, coma, and cachexia | ICD-10: E40.x-E43.x, F00.x-F03.x, G30.x, L89.x, R02.x, R40.2, R64.x  ATC: N06D | NPR,  DNPR |
| Drug misuse | Diagnosis for drug misuse or prescription for drugs used in opioid dependence (in the previous 365 d) | ICD-10: F11.x-F16.x, F18.x, F19.x, T40.x, R78.1-R78.5  ATC: N07BC | NPR,  DNPR |
| Nursing home residence | - | - | NHD |

Abbreviations: CRS, The Civil Registration System; DNPR, The Danish National Prescription Registry; eGFR, estimated glomerular filtration rate; HF, heart failure, ICD-10, International Classification of Diseases 10th Revision; LVEF, left ventricular ejection fraction; MRA, mineralocorticoid receptor antagonist; NCSP, the NOMESCO Classification of Surgical Procedures; NHD, Nursing Home Data; NPR, The National Patient Register; RLRR, The Register of Laboratory Results for Research

Footnote: ^a^ All eligibility criteria were assessed on the index date; unless otherwise stated, the assessment was based on information from the previous ten years

# Supplementary Table S2. Definitions of the primary and secondary outcomes

| Outcome | Definition | Codes | Source |
| --- | --- | --- | --- |
| Primary | | | |
| All-cause mortality | Death due to all causes | N/A | CRS |
| Secondary | | | |
| Cardiovascular death or heart failure hospitalization | Composite outcome based on the first occurrence of cardiovascular mortality or unplanned heart failure hospitalization | ICD-10 (CDR): I00.x-I99.x, R57.0, R96.0, R96.1  ICD-10 (NPR): I11.0, I13.0, I13.2, I42.x, I43.x, I50.x, J81.x, R06.0 | CDR, NPR |
| Cardiovascular death | Cardiovascular causes as underlying cause of death | ICD-10: I00.x-I99.x, R57.0, R96.0, R96.1 | CDR |
| Heart failure hospitalization | Unplanned heart failure hospitalization (primary diagnosis) | ICD-10: I11.0, I13.0, I13.2, I42.x, I43.x, I50.x, J81.x, R06.0 | NPR |

Abbreviations: CDR, The Cause of Death Register; CRS, The Civil Registration System; ICD-10, International Classification of Diseases 10th Revision; NPR, The National Patient Register

# Supplementary Table S3. Definitions of covariates and other baseline characteristics

| Characteristic | Category levels | Codes/Definition | Source |
| --- | --- | --- | --- |
| Demographic characteristics | | | |
| Sex | Female; Male | - | CRS |
| Age (y), mean (SD) ^a^ | N/A | - | CRS |
| Age (y), categorized | 45-49; 50-54; 55-59; 60-64; 65-69; 70-74; 75-79; 80-84;  ≥85 | - | CRS |
| Country of birth | Denmark; Rest of Europe; Outside Europe | - | CRS |
| Civil status | Married/living together; Not married or living together | - | DST |
| Attained education | Primary education; Secondary education or vocational training; Tertiary education; Missing | Highest attained education in the previous y | DST |
| Calendar year ^a^ | 2020; 2021; 2022; 2023; 2024 | - | DNPR |
| Lifestyle health factors | | | |
| Alcohol consumption ^b^ | Below or at recommendations; Above recommendations; Missing | - | DHR |
| Smoking | Never; Current; Past; Missing | - | DHR |
| HF characteristics | | | |
| LVEF (%), mean (SD) ^a^ | N/A | - | DHR |
| LVEF (%), categorized | <25; 25-29; 30-34; 35-40 | - | DHR |
| NYHA-classification | I; II; III; IV; Missing | - | DHR |
| Days since first HF diagnosis, median (IQR) ^a^ | N/A | - | NPR |
| Hospitalized at the time of first HF diagnosis | No; Yes | - | NPR |
| Days since first HF diagnosis, categorized | <90; 90-364; 365-1824; ≥1825 | - | NPR |
| Renal function | | | |
| eGFR (mL/min/1.73 m²), mean (SD) ^a^ | N/A | Based on the most recent eGFR-measurement in the previous 180 d | RLRR |
| eGFR (mL/min/1.73 m²), categorized | >90; 60-89; 45-59; 30-44; 20-29 | Based on the most recent eGFR-measurement in the previous 180 d | RLRR |
| Medical history in the previous 10 years | | | |
| Acute coronary syndrome | No; Yes | ICD-10: I20.0, I21.x, I22.x | NPR |
| Ischemic heart disease | No; Yes | ICD-10: I11.x, I20.x (not I20.0), I23.x-I25.x | NPR |
| Coronary revascularization in the previous year | No; Yes | NCSP: FNA-FNE, FNG0 | NPR |
| Other cardiac surgery or invasive procedure in the previous year | No: Yes | NCSP: F (not FNA-FNE, FNG0), DF020 | NPR |
| Cardiomyopathy | No; Yes | ICD-10: I25.5, I42.x, I43.x | NPR |
| Valve disorder | No; Yes | ICD-10: I34.x-I37.x | NPR |
| Stroke | No; Yes | ICD-10: I60.x-I64.x | NPR |
| Other cerebrovascular disease | No; Yes | ICD-10: G45.x (not G45.4), G46.x, I65.x-I69.x | NPR |
| Atrial fibrillation | No; Yes | ICD-10: I48.x | NPR |
| Other arrythmia | No; Yes | ICD-10: I44.x-I47.x, I49.x | NPR |
| Implantable cardioverter defibrillator | No; Yes | NCSP: BFCB03 | NPR |
| CRT with pacemaker | No; Yes | NCSP: BFCA04, BFCA05, BFCA06 | NPR |
| Peripheral arterial disease (incl. amputation) | No; Yes | ICD-10: E11.5, E13.5, E14.5, I65.x, I70.x, I72.x-I74.x, I77.x, K55.0, K55.1  NCSP: KNFQ, KNGQ, KNHQ | NPR |
| Kidney disease diagnosis | No; Yes | ICD-10: E11.2, E13.2, E14.2, I12.0, I13.1, I13.2, N00.x-N08.x, N10.x-N23.x, N25.x- N29.x | NPR |
| Diabetes complications | No; Yes | ICD-10: E11.0, E11.1, E11.3, E11.4, E11.6-E11.8, E13.0, E13.1, E13.3, E13.4, E13.6-E13.8, E14.0, E14.1, E14.3, E14.4, E14.6-E14.8, E16.0-E16.2, G59.0, G63.2, G99.0, H28.0, H35.8, H36.0, L98.4, M14.2, M14.6, M90.8  NCSP: KCKC10, KCKC12, KCKC15, KCKD65 | NPR |
| COPD | No; Yes | ICD-10: J44.x | NPR |
| Other lung disease | No; Yes | ICD-10: E66.2, I27.x, J40.x-J43.x, J45.x-J47.x, J60.x-J70.x, J84.x, J92.x, J96.x, J98.2, J98.3, R09.2, Z99.x  NCSP: GBB | NPR |
| Venous thromboembolism | No; Yes | ICD-10: I26.x, I80.x (not I80.0), I81.x, I82.0, I82.2-I82.9 | NPR |
| Cancer (excl. non-melanoma skin cancer) | No; Yes | ICD-10: C00.x-C97.x (not C44.x) | NPR |
| Liver disease | No; Yes | ICD-10: B18.x, K70.x-K77.x, I85.0, I85.9, I98.2 | NPR |
| Osteoporosis | No; Yes | ICD-10: M80.x-M82.x  ATC: M05BA, M05BB, M05BX04, M05BX06 | NPR |
| Fracture in the previous y | No; Yes | ICD-10: M48.4, M48.5, M84.3, S02.x (not S02.5), S12.x, S22.x, S32.x, S42.x, S52.x, S62.x, S72.x, S82.x, S92.x, T02.x, T08.x, T10.x, T12.x, | NPR |
| Alcohol-related disorders | No; Yes | ICD-10: E24.4, F10.1-F10.9, G31.2, G62.1, G72.1, I42.6, K29.2, K70.x, K85.2, X65.x  ATC: N07BB | NPR |
| Health care utilization | | | |
| HF hospitalization in the previous 0-29 d | No; Yes | ICD-10: I11.0, I13.0, I13.2,  I42.0, I42.6, I42.7, I42.9, I50.0, I50.1, I50.9 | NPR |
| HF hospitalization in the previous 30-364 d | No; Yes | ICD-10: I11.0, I13.0, I13.2,  I42.0, I42.6, I42.7, I42.9, I50.0, I50.1, I50.9 | NPR |
| Other CV hospitalization in the previous 0-29 d | No; Yes | ICD-10: I00.x-I99.x (not I11.0, I13.0, I13.2, I42.0, I42.6, I42.7, I42.9, I50.0, I50.1, I50.9) | NPR |
| Other CV hospitalization in the previous 30-364 d | No; Yes | ICD-10: I00.x-I99.x (not I11.0, I13.0, I13.2, I42.0, I42.6, I42.7, I42.9, I50.0, I50.1, I50.9) | NPR |
| Other hospitalization in the previous 0-29 d | No; Yes | ICD-10: Not I00.x-I99.x | NPR |
| Other hospitalization in the previous 30-364 d | No; Yes | ICD-10: Not I00.x-I99.x | NPR |
| HF outpatient hospital contact in the previous 0-29 d | No; Yes | ICD-10: I11.0, I13.0, I13.2,  I42.0, I42.6, I42.7, I42.9, I50.0, I50.1, I50.9 | NPR |
| HF outpatient hospital contact in the previous 30-364 d | No; Yes | ICD-10: I11.0, I13.0, I13.2,  I42.0, I42.6, I42.7, I42.9, I50.0, I50.1, I50.9 | NPR |
| Other CV outpatient hospital contact in the previous 0-29 d | No; Yes | ICD-10: I00.x-I99.x (not I11.0, I13.0, I13.2, I42.0, I42.6, I42.7, I42.9, I50.0, I50.1, I50.9) | NPR |
| Other CV outpatient hospital contact in the previous 30-364 d | No; Yes | ICD-10: I00.x-I99.x (not I11.0, I13.0, I13.2, I42.0, I42.6, I42.7, I42.9, I50.0, I50.1, I50.9) | NPR |
| Other outpatient hospital contact in the previous 0-29 d | No; Yes | ICD-10: Not I00.x-I99.x | NPR |
| Other outpatient hospital contact in the previous 30-364 d | No; Yes | ICD-10: Not I00.x-I99.x | NPR |
| Home nursing care in the previous 0-364 d | No; Yes | - | DST |
| Prescription drug use in the previous 365 d | | | |
| Loop diuretic | No; Yes | ATC: C03C, C03EB | DNPR |
| Other diuretic | No; Yes | ATC: C03A, C03B, C03D, C03EA | DNPR |
| ACE-I | No; Yes | ATC: C09A, C09B | DNPR |
| ARB | No; Yes | ATC: C09C, C09D (not C09DX04) | DNPR |
| ARNI | No; Yes | ATC: C09DX04 | DNPR |
| Beta-blocker | No; Yes | ATC: C07 | DNPR |
| SGLT2 inhibitor | No; Yes | ATC: A10BD15, A10BD16,  A10BD19-A10BD21,  A10BD23-A10BD25  A10BK01-A10BK04 | DNPR |
| Calcium-channel blocker | No; Yes | ATC: C08C, C08D | DNPR |
| Digoxin | No; Yes | ATC: C01AA05 | DNPR |
| Nitrate | No; Yes | ATC: C01DA | DNPR |
| Antiarrhythmic drug | No; Yes | ATC: C01B | DNPR |
| Platelet inhibitor | No; Yes | ATC: B01AC | DNPR |
| Anticoagulant | No; Yes | ATC: B01AA, B01AE07, B01AF, B01AX05 | DNPR |
| Lipid lowering drug | No; Yes | ATC: C10 | DNPR |
| Metformin | No; Yes | ATC: A10BA02, A10BD02, A10BD03, A10BD05, A10BD07, A10BD08, A10BD10, A10BD11, A10BD13, A10BD14, A10BD16 | DNPR |
| DPP4 inhibitor | No; Yes | ATC: A10BD07, A10BD08, A10BD09, A10BD10, A10BD11, A10BD13, A10BD24, A10BD25, A10BH01, A10BH02, A10BH03, A10BH04, A10BH05 | DNPR |
| GLP-1 RA | No; Yes | ATC: A10AE54, A10AE56, A10BJ01, A10BJ02, A10BJ03, A10BJ05, A10BJ06 | DNPR |
| Insulin | No; Yes | ATC: A10AB, A10AC, A10AD, A10AE | DNPR |
| Other antidiabetic | No; Yes | ATC: A10BB, A10BD01, A10BD02, A10BD03, A10BD04, A10BD05, A10BD06, A10BD09, A10BD14, A10BF01, A10BG, A10BX | DNPR |
| Antidepressant | No; Yes | ATC: N06A | DNPR |
| Antipsychotic | No; Yes | ATC: N05AA, N05AB, N05AC, N05AD, N05AH, N05AE, N05AF, N05AG, N05AX | DNPR |
| Anxiolytic, hypnotic, or sedative | No; Yes | ATC: N05B, N05C | DNPR |
| Beta-2 agonist inhalant | No; Yes | ATC: R03AC | DNPR |
| Anticholinergic inhalant | No; Yes | ATC: R03BB | DNPR |
| Glucocorticoid inhalant | No; Yes | ATC: R03BA, R03AK | DNPR |
| Oral glucocorticoid | No; Yes | ATC: H02AB | DNPR |
| Opioid | No; Yes | ATC: N02A | DNPR |
| Number of different drugs used in the previous 365 d | 0-9; 10-14; 15-19; ≥20 | - | DNPR |

Abbreviations: ACE-I, Angiotensin-converting-enzyme inhibitor; ARB, Angiotensin II receptor blocker; ARNI, angiotensin receptor-neprilysin inhibitor; ATC, Anatomical Therapeutic Chemical; CKD, chronic kidney disease; COPD, chronic obstructive pulmonary disease; CRS, The Civil Registration System; CRT, cardiac resynchronization therapy; CV, cardiovascular; DNPR, The Danish National Prescription Registry; DHR, The Danish Heart Failure Registry; DPP4, dipeptidyl peptidase 4; DST, Statistics Denmark; eGFR, estimated glomerular filtration rate; GLP-1 RA, glucagon-like peptide-1 receptor agonist; ICD-10, International Classification of Diseases 10th Revision; RLRR, The Register of Laboratory Results for Research; LVEF, left ventricular ejection fraction; MRA, mineralocorticoid receptor antagonist; NCSP, the NOMESCO Classification of Surgical Procedures; NHD, Nursing Home Data; NPR, The National Patient Register; NYHA, New York Heart Association; SGLT2, Sodium-glucose cotransporter-2

Footnotes: ^a^ Not included in propensity score model; ^b^ Recommended limit was defined ≤21 units per week among men and ≤14 units per week among women

# Supplementary Table S4. Subgroup definitions

| Subgroup | Category levels | Codes/Definition | Source |
| --- | --- | --- | --- |
| Sex | Female; Male | N/A | CRS |
| Age (y) | 45-69; ≥70 | N/A | CRS |
| LVEF (%) | <30; 30-40 | N/A | DHR |
| NYHA-class | I-II; III-IV | N/A | DHR |
| Recent HF hospitalization | No; Yes | ICD-10: I11.0, I13.0, I13.2,  I50.x, I42.x, I43.x, J81.x, R06.x (primary diagnosis in the previous 30 days) | NPR |
| Days since first HF diagnosis | 0-89; ≥90 | N/A | NPR |
| History of IHD | No; Yes | ICD-10: I11.x, I20.x-I25.x  NCSP: FNA-FNE, FNG0 | NPR |
| NT-proBNP | Normal/mildly elevated; severely elevated ^a^ | Based on the most recent NT-proBNP-measurement in the previous 180 d | RLRR |
| Chronic kidney disease | No; Yes | CKD was defined as all eGFR measurements in the previous 365 days <60 mL/min/1.73 m² | RLRR |
| Type 2 diabetes | No; Yes | ATC: A10AE54, A10AE56  A10BA02, A10BB, A10BD01-  A10BD11, A10BD13, A10BD14, A10BD16, A10BD24, A10BD25,  A10BF01, A10BG, A10BH01, A10BH02, A10BH03, A10BH04, A10BH05, A10BJ01, A10BJ02, A10BJ03, A10BJ05, A10BJ06,  A10BX  ICD-10: E11.0, E11.1, E11.3, E11.4, E11.6-E11.8, E13.0, E13.1, E13.3, E13.4, E13.6-E13.8, E14.0, E14.1, E14.3, E14.4, E14.6-E14.8, E16.0-E16.2, G59.0, G63.2, G99.0, H28.0, H35.8, H36.0, L98.4, M14.2, M14.6, M90.8  NCSP: KCKC10, KCKC12, KCKC15, KCKD65 | NPR,  DNPR |

Abbreviations: ATC, Anatomical Therapeutic Chemical; CKD, chronic kidney disease; DNPR, The Danish National Prescription Registry; DHR, The Danish Heart Failure Registry; eGFR, estimated glomerular filtration rate; HF, heart failure; ICD-10, International Classification of Diseases 10th Revision; IHD, ischemic heart disease; RLRR, The Register of Laboratory Results for Research; LVEF, left ventricular ejection fraction; NCSP, the NOMESCO Classification of Surgical Procedures; NPR, The National Patient Register; NT-proBNP, N-terminal prohormone of brain natriuretic peptide; NYHA, New York Heart Association
Footnote: ^a^ Normal or mildly elevated NT-proBNP was defined as NT-proBNP≤450 pg/mL for patients aged <50 years, ≤900 pg/mL for those aged 50-74 years, and ≤1800 pg/mL for those aged ≥75 years; severely elevated was defined as NT-proBNP >450 pg/mL for patients aged <50 years, >900 pg/mL for those aged 50-74 years, and >1800 pg/mL for those aged ≥75 years

# Supplementary Table S5. Baseline characteristics before propensity score weighting.

*Values are numbers (percentages) unless otherwise stated.*

| Characteristic | Eplerenone | Spironolactone | SMD |
| --- | --- | --- | --- |
| N | 4550 | 6651 |  |
| Male sex | 3,443 (76) | 4,338 (65) | 0.23 |
| Age (y), mean (SD) ^a^ | 69.5 (10.6) | 70.7 (10.7) |  |
| Age (y), categorized |  |  |  |
| 45-49 | 189 (4) | 217 (3) | 0.05 |
| 50-54 | 291 (6) | 380 (6) | 0.03 |
| 55-59 | 482 (11) | 617 (9) | 0.04 |
| 60-64 | 609 (13) | 772 (12) | 0.05 |
| 65-69 | 664 (15) | 923 (14) | 0.02 |
| 70-74 | 715 (16) | 1,150 (17) | 0.04 |
| 75-79 | 806 (18) | 1,193 (18) | 0.01 |
| 80-84 | 550 (12) | 877 (13) | 0.03 |
| ≥85 | 244 (5) | 522 (8) | 0.10 |
| Country of birth |  |  |  |
| Denmark | 4,237 (93) | 6,131 (92) | 0.04 |
| Rest of Europe | 214 (5) | 335 (5) | 0.02 |
| Outside Europe | 99 (2) | 185 (3) | 0.04 |
| Civil status |  |  |  |
| Not married | 2,035 (45) | 3,232 (49) | 0.08 |
| Married | 2,515 (55) | 3,419 (51) | 0.08 |
| Attained education |  |  |  |
| Primary school | 1,376 (30) | 2,251 (34) | 0.08 |
| Secondary school and vocational training | 2,117 (47) | 2,910 (44) | 0.06 |
| Tertiary education | 781 (17) | 1,103 (17) | 0.02 |
| Education: Missing | 276 (6) | 387 (6) | 0.01 |
| Calendar year ^a^ |  |  |  |
| 2020 | 485 (11) | 1,405 (21) |  |
| 2021 | 864 (19) | 1,429 (21) |  |
| 2022 | 1,132 (25) | 1,234 (19) |  |
| 2023 | 1,341 (29) | 1,530 (23) |  |
| 2024 | 728 (16) | 1,053 (16) |  |
| Lifestyle health factors |  |  |  |
| Alcohol consumption ^b^ | 3,877 (85) | 5,775 (87) | 0.05 |
| Below or at recommendations | 569 (13) | 706 (11) | 0.06 |
| Above recommendations | 104 (2) | 170 (3) | 0.02 |
| Missing |  |  |  |
| Smoking |  |  |  |
| Never | 1,547 (34) | 2,200 (33) | 0.02 |
| Current | 1,073 (24) | 1,699 (26) | 0.05 |
| Past | 1,863 (41) | 2,609 (39) | 0.04 |
| Missing | 67 (1) | 143 (2) | 0.05 |
| HF characteristics |  |  |  |
| LVEF (%), mean (SD) ^a^ | 28.9 (8.4) | 28.6 (8.4) |  |
| LVEF (%), categorized |  |  |  |
| 35-40 | 1,668 (37) | 2,348 (35) | 0.03 |
| 30-34 | 950 (21) | 1,376 (21) | 0.00 |
| 25-29 | 686 (15) | 1,014 (15) | 0.00 |
| <25 | 1,246 (27) | 1,913 (29) | 0.03 |
| NYHA-classification |  |  |  |
| I | 526 (12) | 860 (13) | 0.04 |
| II | 3,309 (73) | 4,606 (69) | 0.08 |
| III | 670 (15) | 1,097 (16) | 0.05 |
| IV | 32 (1) | 43 (1) | 0.01 |
| Missing | 13 (0) | 45 (1) | 0.06 |
| Hospitalized at the time of first HF-diagnosis | 2,175 (48) | 3,495 (53) | 0.10 |
| Days since first HF diagnosis, median (IQR) ^a^ | 51.0 (15.0-134.0) | 55.0 (10.0-174.0) |  |
| Days since first HF diagnosis, categorized |  |  |  |
| <90 | 2,949 (65) | 4,086 (61) | 0.07 |
| 90-179 | 719 (16) | 929 (14) | 0.05 |
| 180-364 | 309 (7) | 408 (6) | 0.03 |
| ≥365 | 573 (13) | 1,228 (18) | 0.16 |
| Renal function |  |  |  |
| eGFR (mL/min/1.73 m²), mean (SD) ^a^ | 73.4 (17.0) | 72.2 (17.6) |  |
| eGFR (mL/min/1.73 m²), categorized |  |  |  |
| ≥90 | 905 (20) | 1,236 (19) | 0.03 |
| 60-89 | 2,568 (56) | 3,636 (55) | 0.04 |
| 45-59 | 859 (19) | 1,303 (20) | 0.02 |
| 30-44 | 191 (4) | 414 (6) | 0.09 |
| Missing | 27 (1) | 62 (1) | 0.04 |
| Medical history in the previous 10 y |  |  |  |
| Acute coronary syndrome | 927 (20) | 1,330 (20) | 0.01 |
| Ischemic heart disease | 1,349 (30) | 1,965 (30) | 0.00 |
| Coronary revascularization in the previous year | 740 (16) | 904 (14) | 0.08 |
| Other cardiac surgery or invasive procedure in the previous y | 216 (5) | 246 (4) | 0.05 |
| Cardiomyopathy | 518 (11) | 778 (12) | 0.01 |
| Valve disorder | 427 (9) | 640 (10) | 0.01 |
| Stroke | 352 (8) | 482 (7) | 0.02 |
| Other cerebrovascular disease | 291 (6) | 446 (7) | 0.01 |
| Atrial fibrillation | 1,492 (33) | 2,177 (33) | 0.00 |
| Other arrythmia | 826 (18) | 1,219 (18) | 0.00 |
| Implantable cardioverter defibrillator | 84 (2) | 109 (2) | 0.02 |
| CRT with pacemaker | 25 (1) | 59 (1) | 0.04 |
| Peripheral arterial disease | 413 (9) | 618 (9) | 0.01 |
| Kidney disease diagnosis | 319 (7) | 519 (8) | 0.03 |
| Diabetes complications | 381 (8) | 546 (8) | 0.01 |
| COPD | 371 (8) | 705 (11) | 0.08 |
| Other lung disease | 436 (10) | 758 (11) | 0.06 |
| Venous thromboembolism | 228 (5) | 358 (5) | 0.02 |
| Cancer (excl. non-melanoma skin cancer) | 633 (14) | 940 (14) | 0.01 |
| Liver disease | 88 (2) | 152 (2) | 0.02 |
| Osteoporosis | 411 (9) | 709 (11) | 0.05 |
| Fracture in the previous year | 148 (3) | 214 (3) | 0.00 |
| Alcohol-related disorders | 140 (3) | 209 (3) | 0.00 |
| Health care utilization |  |  |  |
| HF hospitalization in the previous 0-29 d | 1,285 (28) | 2,101 (32) | 0.07 |
| HF hospitalization in the previous 30-364 d | 1,273 (28) | 1,581 (24) | 0.10 |
| Other CV hospitalization in the previous 0-29 d | 568 (12) | 782 (12) | 0.02 |
| Other CV hospitalization in the previous 30-364 d | 1,490 (33) | 1,749 (26) | 0.14 |
| Other hospitalization in the previous 0-29 d | 685 (15) | 1,222 (18) | 0.09 |
| Other hospitalization in the previous 30-364 d | 1,980 (44) | 2,827 (43) | 0.02 |
| HF outpatient hospital contact in the previous 0-29 d | 3,282 (72) | 4,065 (61) | 0.24 |
| HF outpatient hospital contact in the previous 30-364 d | 2,181 (48) | 2,748 (41) | 0.13 |
| Other CV outpatient hospital contact in the previous 0-29 d | 620 (14) | 718 (11) | 0.09 |
| Other CV outpatient hospital contact in the previous 30-364 d | 1,277 (28) | 1,519 (23) | 0.12 |
| Other outpatient hospital contact in the previous 0-29 d | 1,773 (39) | 2,508 (38) | 0.03 |
| Other outpatient hospital contact in the previous 30-364 d | 3,365 (74) | 4,805 (72) | 0.04 |
| Home nursing care in the previous 365 d | 940 (21) | 1,598 (24) | 0.08 |
| Prescription drug use in the previous 365 d |  |  |  |
| Loop diuretic | 3,030 (67) | 4,506 (68) | 0.02 |
| Other diuretic | 442 (10) | 797 (12) | 0.07 |
| ACEi | 2,945 (65) | 3,854 (58) | 0.14 |
| ARB | 1,383 (30) | 2,067 (31) | 0.01 |
| ARNI | 290 (6) | 293 (4) | 0.09 |
| Beta-blocker | 3,918 (86) | 5,589 (84) | 0.06 |
| SGLT2-inhibitor | 2,149 (47) | 2,423 (36) | 0.22 |
| Calcium-channel blocker | 1,058 (23) | 1,619 (24) | 0.03 |
| Digoxin | 408 (9) | 740 (11) | 0.07 |
| Nitrate | 514 (11) | 843 (13) | 0.04 |
| Antiarrhythmic drugs | 387 (9) | 418 (6) | 0.08 |
| Platelet inhibitors | 2,170 (48) | 2,973 (45) | 0.06 |
| Anticoagulants | 1,856 (41) | 2,719 (41) | 0.00 |
| Lipid lowering drug | 2,829 (62) | 3,945 (59) | 0.06 |
| Metformin | 738 (16) | 1,087 (16) | 0.00 |
| DPP4 inhibitor | 112 (2) | 169 (3) | 0.01 |
| GLP-1 RA | 258 (6) | 354 (5) | 0.02 |
| Insulin | 275 (6) | 350 (5) | 0.03 |
| Other antidiabetic | 55 (1) | 81 (1) | 0.00 |
| Antidepressant | 541 (12) | 899 (14) | 0.05 |
| Antipsychotic | 118 (3) | 195 (3) | 0.02 |
| Anxiolytic, hypnotic, or sedative | 518 (11) | 899 (14) | 0.06 |
| Beta-2 agonist inhalant | 771 (17) | 1,246 (19) | 0.05 |
| Anticholinergic inhalant | 159 (3) | 318 (5) | 0.06 |
| Glucocorticoid inhalant | 466 (10) | 733 (11) | 0.03 |
| Oral glucocorticoid | 432 (9) | 756 (11) | 0.06 |
| Opioid | 729 (16) | 1,095 (16) | 0.01 |
| Number of different drugs used in the previous 365 d |  |  |  |
| <10 | 1,527 (34) | 2,258 (34) | 0.01 |
| 10-14 | 1,839 (40) | 2,562 (39) | 0.04 |
| 15-19 | 835 (18) | 1,305 (20) | 0.03 |
| ≥20 | 349 (8) | 526 (8) | 0.01 |

Abbreviations: COPD, chronic obstructive pulmonary disease; CRT, cardiac resynchronization therapy; CV, cardiovascular; DPP4, dipeptidyl peptidase 4; eGFR, estimated glomerular filtration rate; GLP-1 RA, glucagon-like peptide-1 receptor agonist; LVEF, left ventricular ejection fraction; MRA, mineralocorticoid receptor antagonist; NYHA, New York Heart Association; SGLT2, sodium-glucose co-transporter 2; SMD, standardize mean difference

Footnotes: ^a^ Not included in propensity score model; ^b^ Recommended limit was defined as ≤21 units per week among men and ≤14 units per week among women

# Supplementary Table S6. Odds ratios from the propensity score model

| Characteristic | Odds ratio (95% CI) |
| --- | --- |
| N |  |
| Male sex | 1.61 (1.46-1.77) |
| Age (y), categorized |  |
| 45-49 | 1.00 (REF) |
| 50-54 | 0.88 (0.68-1.13) |
| 55-59 | 0.85 (0.67-1.07) |
| 60-64 | 0.85 (0.68-1.08) |
| 65-69 | 0.79 (0.63-1.00) |
| 70-74 | 0.68 (0.54-0.86) |
| 75-79 | 0.75 (0.59-0.95) |
| 80-84 | 0.76 (0.59-0.97) |
| ≥85 | 0.67 (0.51-0.88) |
| Country of birth |  |
| Denmark | 1.00 (REF) |
| Rest of Europe | 0.94 (0.78-1.14) |
| Outside Europe | 0.73 (0.57-0.95) |
| Civil status |  |
| Not married | 1.00 (REF) |
| Married | 1.08 (0.99-1.17) |
| Attained education |  |
| Primary school | 1.00 (REF) |
| Secondary school and vocational training | 1.06 (0.96-1.16) |
| Tertiary education | 1.04 (0.92-1.17) |
| Missing | 1.06 (0.89-1.27) |
| Lifestyle health factors |  |
| Alcohol consumption ^a^ |  |
| Below or at recommendations | 1.00 (REF) |
| Above recommendations | 1.13 (0.99-1.28) |
| Missing | 1.32 (0.98-1.78) |
| Smoking |  |
| Never | 1.00 (REF) |
| Current | 0.87 (0.78-0.97) |
| Past | 0.99 (0.90-1.09) |
| Missing | 0.65 (0.46-0.93) |
| HF characteristics |  |
| LVEF (%), categorized |  |
| 35-40 | 1.00 (REF) |
| 30-34 | 0.98 (0.88-1.09) |
| 25-29 | 0.95 (0.84-1.07) |
| <25 | 0.90 (0.81-1.01) |
| NYHA-classification |  |
| I | 1.00 (REF) |
| II | 1.22 (1.07-1.38) |
| III | 1.15 (0.98-1.35) |
| IV | 1.37 (0.84-2.23) |
| Missing | 0.78 (0.41-1.49) |
| Hospitalized at the time of first HF-diagnosis | 0.90 (0.81-0.99) |
| Days since first HF diagnosis, categorized |  |
| <90 | 1.00 (REF) |
| 90-179 | 0.85 (0.75-0.97) |
| 180-364 | 0.84 (0.71-1.00) |
| ≥365 | 0.69 (0.60-0.79) |
| Renal function |  |
| eGFR (mL/min/1.73 m²), categorized |  |
| ≥90 | 1.00 (REF) |
| 60-89 | 1.03 (0.92-1.16) |
| 45-59 | 1.05 (0.91-1.21) |
| 30-44 | 0.84 (0.67-1.04) |
| Missing | 1.15 (0.71-1.87) |
| Medical history in the previous 10 years |  |
| Acute coronary syndrome | 0.92 (0.81-1.05) |
| Ischemic heart disease | 0.97 (0.88-1.07) |
| Coronary revascularization in the previous year | 0.93 (0.80-1.09) |
| Other cardiac surgery or invasive procedure in the previous y | 1.03 (0.83-1.27) |
| Cardiomyopathy | 1.06 (0.93-1.20) |
| Valve disorder | 0.92 (0.80-1.06) |
| Stroke | 1.05 (0.89-1.24) |
| Other cerebrovascular disease | 0.94 (0.79-1.12) |
| Atrial fibrillation | 1.03 (0.90-1.18) |
| Other arrythmia | 0.94 (0.85-1.05) |
| Implantable cardioverter defibrillator | 1.08 (0.80-1.47) |
| CRT with pacemaker | 0.65 (0.40-1.06) |
| Peripheral arterial disease | 1.00 (0.87-1.16) |
| Kidney disease diagnosis | 0.89 (0.77-1.04) |
| Diabetes complications | 0.98 (0.83-1.16) |
| COPD | 0.92 (0.78-1.08) |
| Other lung disease | 0.94 (0.81-1.08) |
| Venous thromboembolism | 0.97 (0.81-1.16) |
| Cancer (excl. non-melanoma skin cancer) | 1.03 (0.92-1.16) |
| Liver disease | 0.95 (0.71-1.26) |
| Osteoporosis | 1.07 (0.93-1.24) |
| Fracture in the previous year | 1.10 (0.87-1.39) |
| Alcohol-related disorders | 1.02 (0.81-1.30) |
| Health care utilization |  |
| HF hospitalization in the previous 0-29 d | 1.09 (0.98-1.21) |
| HF hospitalization in the previous 30-364 d | 1.12 (1.01-1.25) |
| Other CV hospitalization in the previous 0-29 d | 1.16 (1.01-1.32) |
| Other CV hospitalization in the previous 30-364 d | 1.11 (0.99-1.24) |
| Other hospitalization in the previous 0-29 d | 0.93 (0.83-1.04) |
| Other hospitalization in the previous 30-364 d | 1.01 (0.92-1.10) |
| HF outpatient hospital contact in the previous 0-29 d | 1.40 (1.26-1.55) |
| HF outpatient hospital contact in the previous 30-364 d | 1.07 (0.96-1.18) |
| Other CV outpatient hospital contact in the previous 0-29 d | 1.30 (1.14-1.48) |
| Other CV outpatient hospital contact in the previous 30-364 d | 1.16 (1.04-1.28) |
| Other outpatient hospital contact in the previous 0-29 d | 1.06 (0.98-1.16) |
| Other outpatient hospital contact in the previous 30-364 d | 1.03 (0.93-1.14) |
| Home nursing care in the previous 365 d | 0.97 (0.87-1.08) |
| Prescription drug use in the previous 365 d |  |
| Loop diuretic | 1.05 (0.95-1.16) |
| Other diuretic | 0.86 (0.75-0.98) |
| ACEi | 1.21 (1.09-1.35) |
| ARB | 1.09 (0.98-1.22) |
| ARNI | 1.41 (1.18-1.69) |
| Beta-blocker | 1.04 (0.93-1.17) |
| SGLT2-inhibitor | 1.51 (1.39-1.64) |
| Calcium-channel blocker | 0.93 (0.84-1.02) |
| Digoxin | 0.79 (0.68-0.92) |
| Nitrate | 0.82 (0.72-0.94) |
| Antiarrhythmic drugs | 1.25 (1.06-1.47) |
| Platelet inhibitors | 1.09 (0.98-1.22) |
| Anticoagulants | 0.94 (0.83-1.07) |
| Lipid lowering drug | 1.04 (0.94-1.15) |
| Metformin | 0.90 (0.79-1.02) |
| DPP4 inhibitor | 0.98 (0.75-1.28) |
| GLP-1 RA | 0.92 (0.75-1.11) |
| Insulin | 1.19 (0.97-1.46) |
| Other antidiabetic | 0.95 (0.65-1.37) |
| Antidepressant | 0.95 (0.83-1.08) |
| Antipsychotic | 0.98 (0.76-1.26) |
| Anxiolytic, hypnotic, or sedative | 0.90 (0.79-1.02) |
| Beta-2 agonist inhalant | 0.99 (0.87-1.12) |
| Anticholinergic inhalant | 0.85 (0.68-1.06) |
| Glucocorticoid inhalant | 1.07 (0.92-1.24) |
| Oral glucocorticoid | 0.88 (0.76-1.02) |
| Opioid | 1.06 (0.94-1.19) |
| Number of different drugs used in the previous 365 d |  |
| <10 | 1.00 (REF) |
| 10-14 | 1.03 (0.92-1.15) |
| 15-19 | 1.00 (0.85-1.17) |
| ≥20 | 1.09 (0.86-1.38) |

Abbreviations: ACE-I, Angiotensin-converting-enzyme inhibitor; ARB, Angiotensin II receptor blocker; ARNI, angiotensin receptor-neprilysin inhibitor; ATC, Anatomical Therapeutic Chemical; COPD, chronic obstructive pulmonary disease; CRT, cardiac resynchronization therapy; CV, cardiovascular; DPP4, dipeptidyl peptidase 4; eGFR, estimated glomerular filtration rate; GLP-1 RA, glucagon-like peptide-1 receptor agonist; LVEF, left ventricular ejection fraction; MRA, mineralocorticoid receptor antagonist; NYHA, New York Heart Association
Footnotes: ^a^ Recommended limit was defined as ≤21 units per week among men and ≤14 units per week among women

# Supplementary Figure S1. Weighted incidence curve for treatment discontinuation in the analysis of the primary outcome


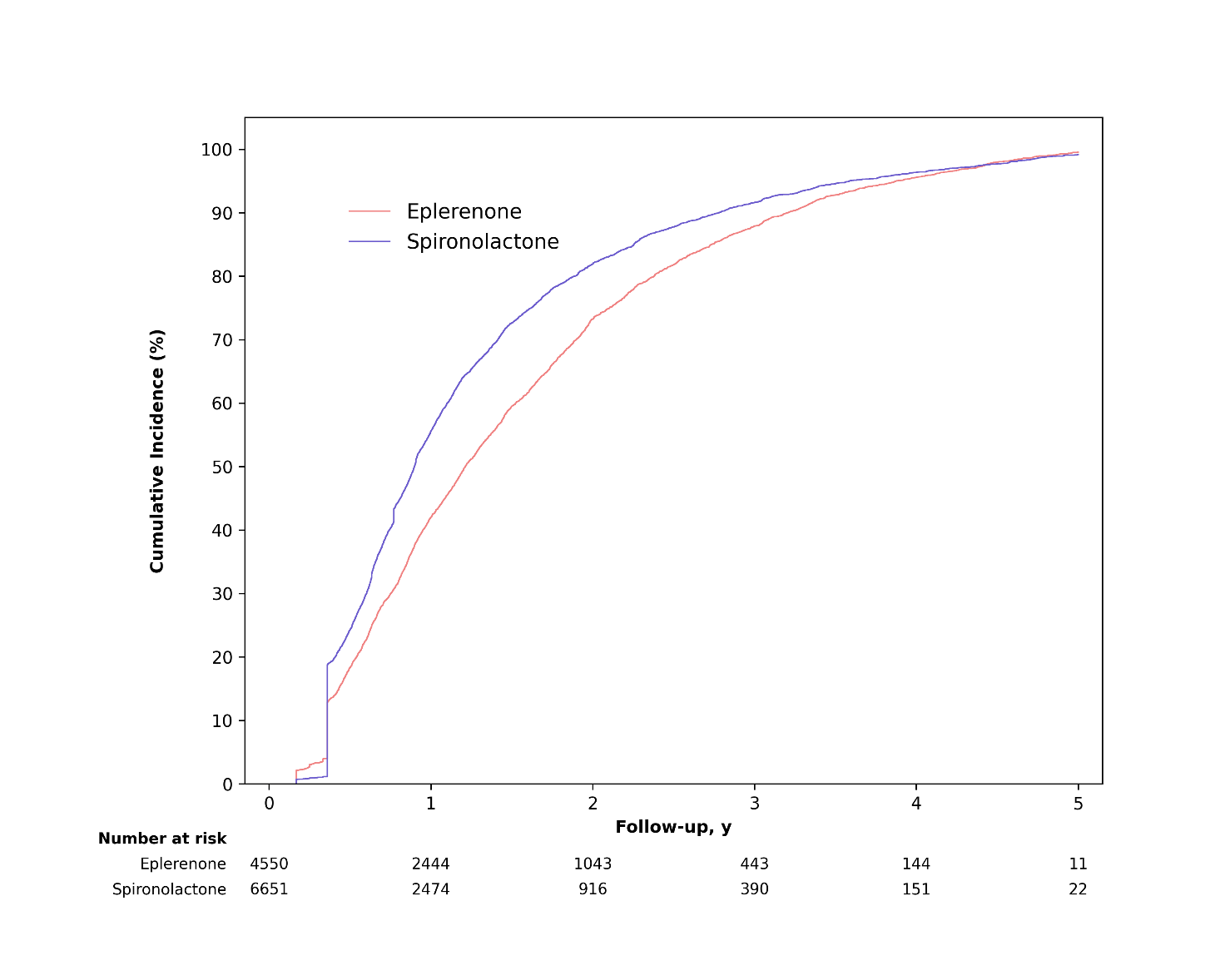


# Supplementary Figure S2. Weighted cumulative incidence curve for cardiovascular death or heart failure hospitalization


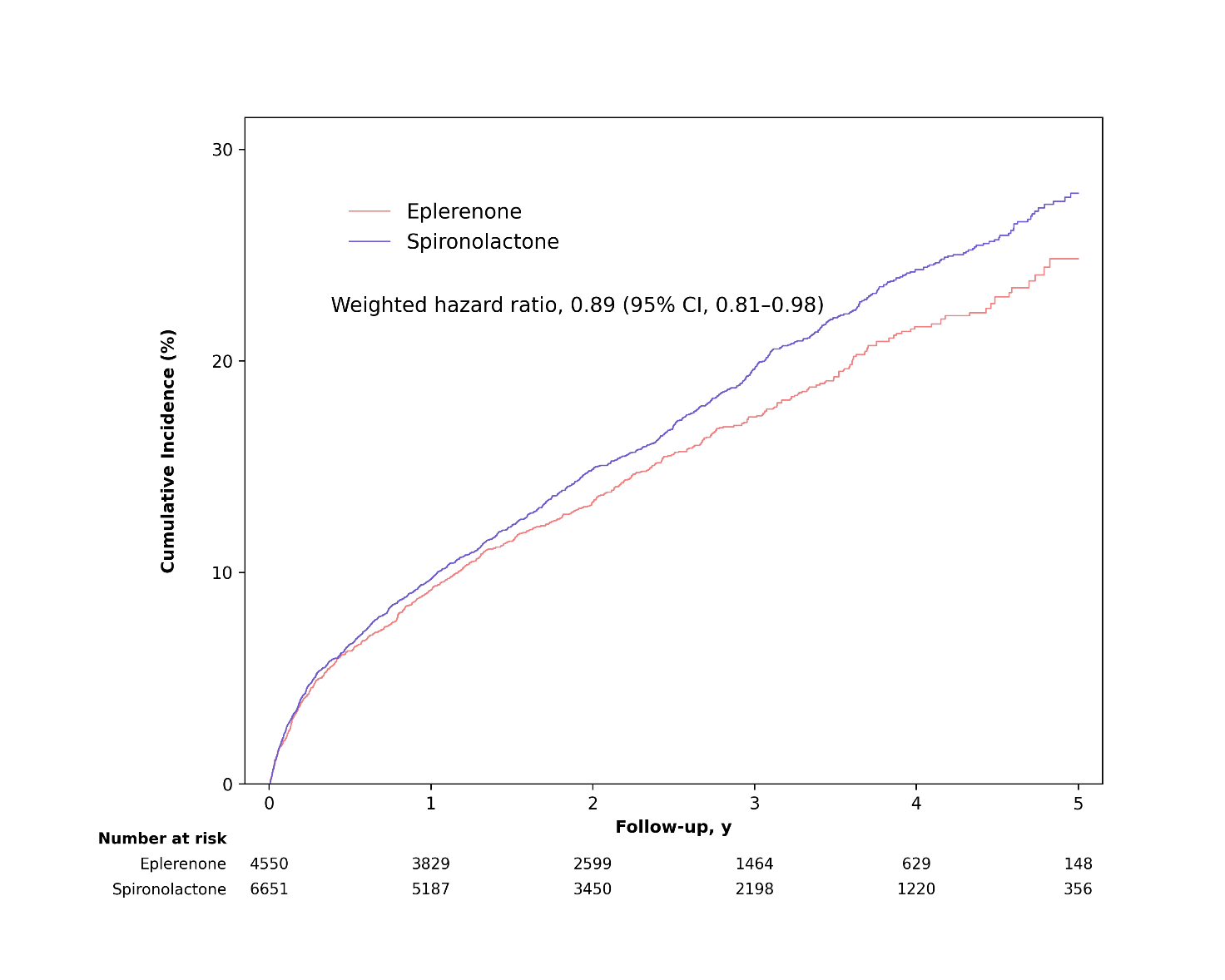


# Supplementary Figure S3. Weighted cumulative incidence curve for cardiovascular death


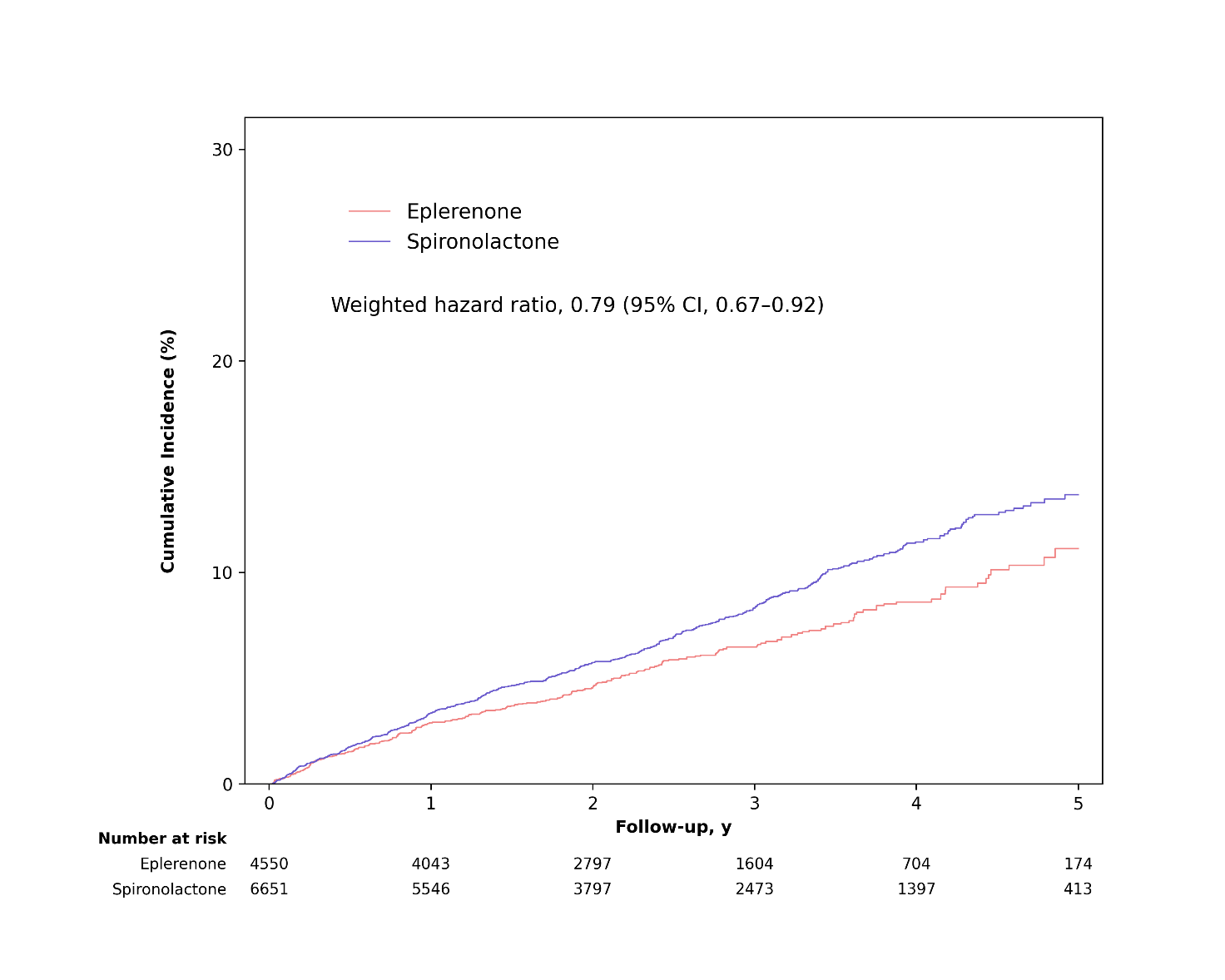


# Supplementary Figure S4. Weighted cumulative incidence curve for heart failure hospitalization


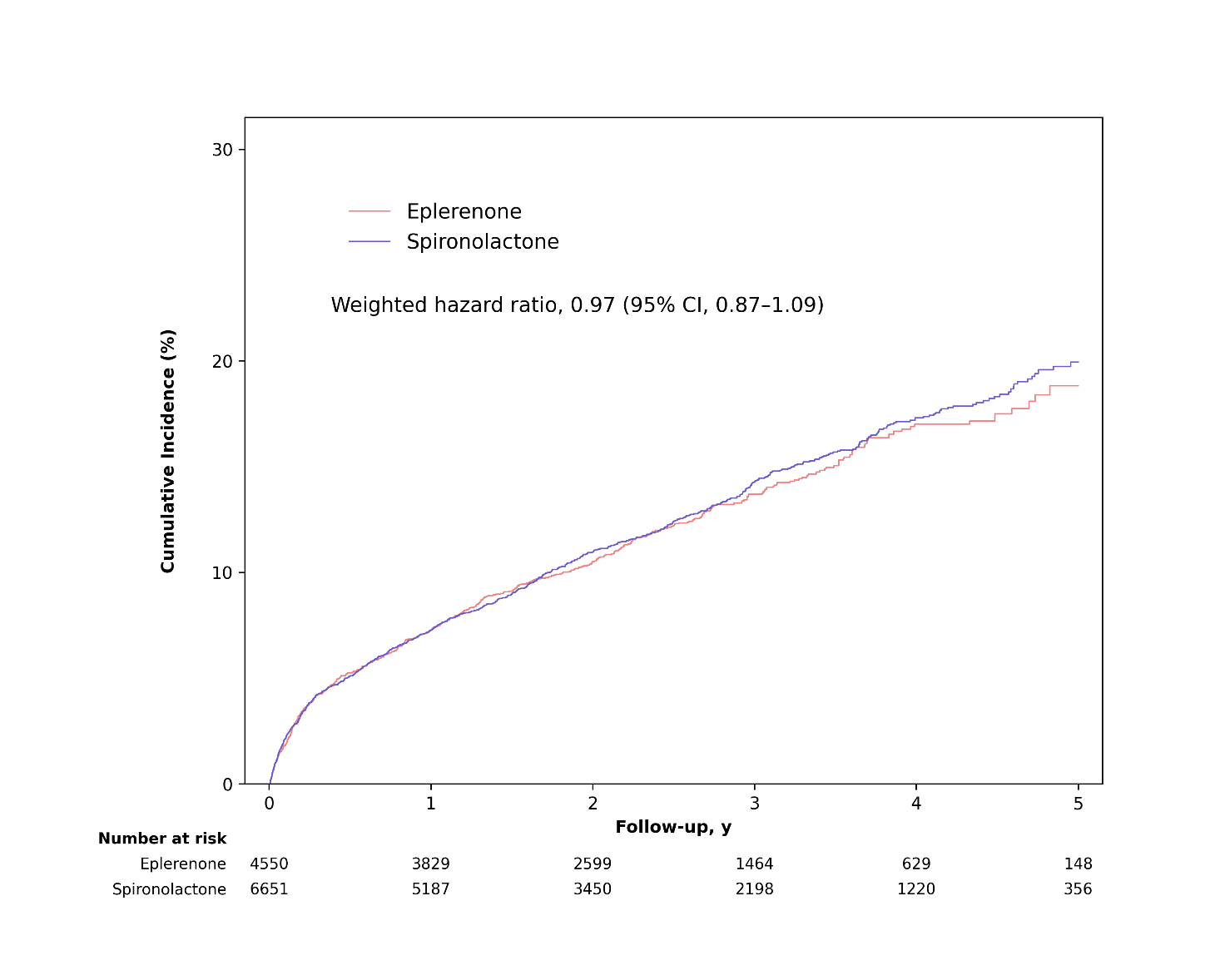


# Supplementary Table S7. Complementary analyses of the primary outcome

| Sensitivity analysis | Eplerenone | | | Spironolactone | | | IPTW-weighted HR (95% CI) |
| --- | --- | --- | --- | --- | --- | --- | --- |
|  | N | Events | Rate per 100 p-y | N | Events | Rate per 100 p-y |  |
| Per-protocol analysis | 4550 | 258 | 4.1 | 6651 | 385 | 5.4 | 0.96 (0.81-1.13) |
| ITT without censoring at crossover | 4550 | 606 | 5.2 | 6651 | 1219 | 6.6 | 0.91 (0.83-1.01) |

Abbreviations: CI, confidence interval; HR, hazard ratio; IPTW, inverse‐probability‐of‐treatment weighting; ITT, Intention-to-treat; MRA, mineralocorticoid receptor antagonists; p-y, person-years

# Supplementary Figure S5. Incidence curve for the primary outcome using per-protocol analysis


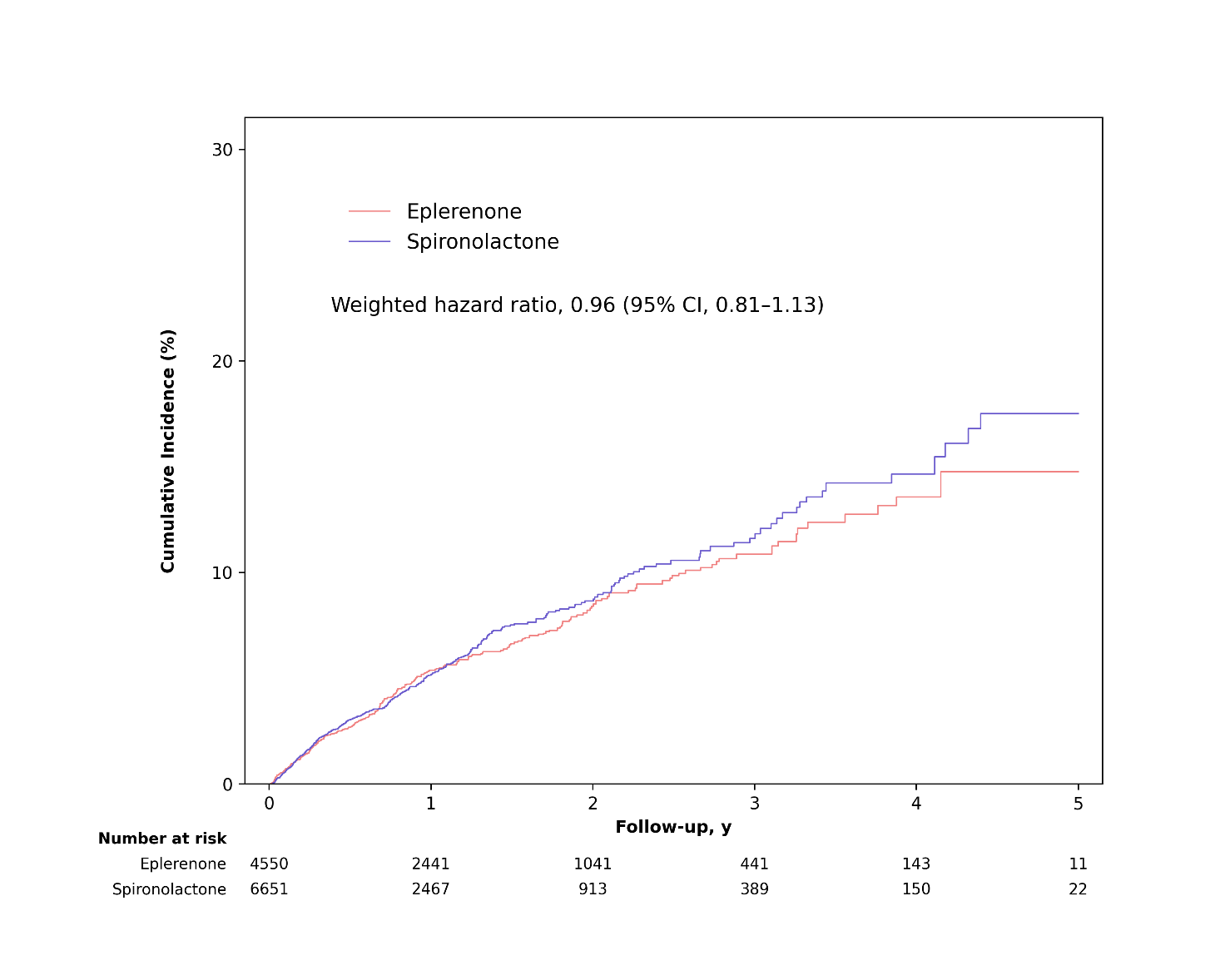

Supplement: pvag030_Supplementary_Data [file pvag030_supplementary_data.docx]
